# On the Definition of Energy Flux in One-Dimensional Chains of Particles

**DOI:** 10.3390/e21111036

**Published:** 2019-10-25

**Authors:** Paolo De Gregorio

**Affiliations:** Dipartimento di Scienze Matematiche, Politecnico di Torino, Corso Duca degli Abruzzi 24, I-10129 Torino, Italy; paolo.degregorio@polito.it; Tel.: +39-011-090-7538

**Keywords:** energy flux, heat flux, Fermi-Pasta-Ulam-Tsingou chain, nonequilibrium steady states, dissipative systems, transport properties

## Abstract

We review two well-known definitions present in the literature, which are used to define the heat or energy flux in one dimensional chains. One definition equates the energy variation per particle to a discretized flux difference, which we here show it also corresponds to the flux of energy in the zero wavenumber limit in Fourier space, concurrently providing a general formula valid for all wavelengths. The other relies somewhat elaborately on a definition of the flux, which is a function of every coordinate in the line. We try to shed further light on their significance by introducing a novel integral operator, acting over movable boundaries represented by the neighboring particles’ positions, or some combinations thereof. By specializing to the case of chains with the particles’ order conserved, we show that the first definition corresponds to applying the differential continuity-equation operator after the application of the integral operator. Conversely, the second definition corresponds to applying the introduced integral operator to the energy flux. It is, therefore, an integral quantity and not a local quantity. More worryingly, it does not satisfy in any obvious way an equation of continuity. We show that in stationary states, the first definition is resilient to several formally legitimate modifications of the (models of) energy density distribution, while the second is not. On the other hand, it seems peculiar that this integral definition appears to capture a transport contribution, which may be called of convective nature, which is altogether missed by the former definition. In an attempt to connect the dots, we propose that the locally integrated flux divided by the inter-particle distance is a good measure of the energy flux. We show that the proposition can be explicitly constructed analytically by an ad hoc modification of the chosen model for the energy density.

## 1. Introduction

After the advent of computer numerical simulations [1], one-dimensional models have represented the benchmark for studying several basic principles of statistical mechanics, as well as understanding the mathematical foundations of some equilibrium thermodynamical properties [1,2,3,4,5,6,7,8,9,10,11,12,13]. After the earlier studies on equilibrium thermodynamics, in relatively recent years they have been exploited in the field of nonequilibrium statistical mechanics. A typical field of study is that of nonequilibrium stationary states (NESS) [14,15,16,17,18], or eventually transient states [19,20], when a temperature gradient is present at the line’s boundaries. The temperature is usually identified with the kinetic temperature, i.e., it is assumed to be proportional to the variance of the velocity. This is achieved through so-called numerical thermostats, which can be deterministic or stochastic. The interaction between the bulk particles is typically Newtonian.

One-dimensional systems are characteristically anomalous models for heat conduction and transport [21,22,23,24,25,26,27,28,29]. One related problem is defining the observable that can be identified with the heat flux. The heat flux may be thought of as the energy flux, net of collective coherent motions. Even at the level of energy flux, there has been some concern as to what its precise definition should be, in function of the microscopic variables [14,30,31,32,33,34,35]. In fact, in real physical systems, the energy or the heat flux can be measured over mesoscopic regions of space. In numerical simulations, one monitors the dynamical evolution of individual particles, and the size of the system may be small compared to real physical systems. One ends up defining the energy flux particle-by-particle, thus implying the existence of a measurable quantity which is microscopic in nature. The fact that the defined flux is, on average, constant over all the particles in NESS is a loose indicator that it is a good candidate for being identified as the real physical heat flux.

It has emerged recently that the density profile of several one-dimensional models is not constant along the chain. Rather, the inverse density is linearly related to the kinetic temperature profile [36]. This poses some additional concern over the viability of the known definitions of energy flux, if not identified correctly, as a flux which is uniform on average in terms of particle-by-particle measurements may not appear so when considered in coordinate space. In fact, in a densely populated region, the same energy may, in principle, be transferred in a smaller region than that which is transferred in a region of low density. Assuming one has correctly identified the observable energy flux in a NESS, this should be constant everywhere. On the other hand, as more particles participate to the flow in a high density region than in a low density region, the behavior of the fluctuations of the flow cannot be freely anticipated.

To put things into their proper context, consider the following adaptation to one dimension of the derivation of the continuity equation,

(1) $$\frac{d}{dt}\int_{a}^{b}h\left(x,t\right)\;\;d\;\;x=\phi \left(a,t\right)-\phi \left(b,t\right)\;,$$

where h(x,t) is the energy density at the point *x* at time *t*. *a* and *b* are two fixed points internal to the line where the energy is flowing and ϕ(a,t)−ϕ(b,t) the instantaneous in-flow of energy minus the out-flow of energy at the boundaries. Then,

(2) $$\int_{a}^{b}\frac{\partial h(x,t)}{\partial t}\;\;d\;\;x=-\int_{a}^{b}\frac{\partial \phi (x,t)}{\partial x}\;\;d\;\;x\;,$$

and per de arbitrariness of *a* and *b*, it follows that,

(3) $$\frac{\partial h(x,t)}{\partial t}+\frac{\partial \phi (x,t)}{\partial x}=0\;.$$

The continuity equation, therefore, is usually seen as a consequence of the condition that the energy contained between fixed points varies in time via flux differences established at those points. Conversely, here we assume that it is valid a priori. The fixed space coordinates are thus the ideal referential points for monitoring the flow. However, in one-dimensional models, often the particles’ positions and momenta are the theory’s reference points, since they appear natural and are monitored in simulations. The contribution to the flux between *a* and *b* comes from both the change due to the interactions between the particles inside the segment and to the transport of particles inside to outside the segment. We refer to “conduction” for the first type, and to “convection” for the second type.

Observe that given any arbitrary laws (a(t),b(t)), then,

(4) $$\int_{a(t)}^{b(t)}\frac{\partial \phi (x,t)}{\partial x}\;\;d\;\;x=\phi \left(b\left(t\right),t\right)-\phi \left(a\left(t\right),t\right)\;,$$

but

(5) $$\frac{d}{dt}\int_{a(t)}^{b(t)}h\left(x,t\right)\;\;d\;\;x\neq \phi \left(a\left(t\right),t\right)-\phi \left(b\left(t\right),t\right)\;,$$

and

(6) $$\frac{d}{dt}\int_{a(t)}^{b(t)}h\left(x,t\right)\;\;d\;\;x\neq \int_{a(t)}^{b(t)}\frac{\partial h(x,t)}{\partial t}\;\;d\;\;x\;.$$

The equality holds in place of the inequation only in some special circumstances. For example, following Leibniz’s integral rule, one sufficient condition is that h(a(t);t)=h(b(t),t)=0 for any *t* in the time interval of interest. Notice, however, that often the energy density is assumed to be (singularly) concentrated at the particles’ positions. In the hypothesis that h(x,t) is almost surely zero everywhere on the line, then the equality holds almost surely, unless (a(t),b(t)) is chosen in a very peculiar manner.

The above observations are pertinent for simulations studies, as the observables are the positions and momenta of the particles, and therefore the evolution in time of the energy is not usually rendered explicit in terms of fixed coordinates on the line. Although the real physical energy is spatially extended (the electromagnetic field), in classical simulations, it is customary yet fully effective to assign the energy to the particles themselves. The traditional view is that two definitions of energy flux coexist [34], one called “discrete”, and one “continuous”. Continuous does not mean that the flux is a continuous function, but rather that its spatial domain is an interval on the real line, whereas the discrete definition has a closed set on natural numbers as domain (the indexed particles).

As we shall see in this work, one can put the two definitions in a closer relation by considering special integral operators. The implication is that the considered quantities are integrals of the mentioned type, with a(t) and b(t) the coordinates of neighboring particles (precisely the mentioned peculiar choices). This is at the root of some of the difficulties in agreeing on the definition of energy or heat flux that must be assumed. As we shall see, specifically for the case of a linear chain with conserved order of particles, it is in fact possible to define a function $\phi_{n}\left(t\right)$, such that

(7) $$\frac{d}{dt}\int_{x_{n}\left(t\right)}^{x_{n+1}\left(t\right)}h\left(x,t\right)\;\;d\;\;x=\phi_{n}\left(t\right)-\phi_{n+1}\left(t\right)\;,$$

where $x_{n}\left(t\right)$ is the position of the *n*-th particle. Clearly, $\phi_{n}\left(t\right)$ is indeed a flux variable, which, however, should not be confused with $\phi (x_{n}\left(t\right),t)$, per the inequation $\frac{d}{dt}\int_{a(t)}^{b(t)}h\left(x,t\right)\;\;d\;\;x\neq \phi \left(a\left(t\right),t\right)-\phi \left(b\left(t\right),t\right)$.

As just mentioned, a special situation is that in which h(x(t),t)=0 for x(t) a given trajectory on the line. This is also problematic, since the particles populate the line and one may argue, with some legitimacy, that nowhere does the energy density equate zero. However, the most common definitions (i.e., models) of the (continuous) energy density precisely assume that the energy density is pointwise accumulated at the positions of the particles, in the form of a combination of Dirac’s delta functions [32,33,34]. Other models of energy density would be legitimate, but the results of the theory should not depend on such freedom.

Finally, propositions have been made to capture the convective contribution to the heat flux for models that move away from quasi crystalline states [33]. As we shall see, but as yet not recognized, this step involves considering the following integrated quantity,

(8) $$j_{n}\left(t\right)=\int_{x_{n}\left(t\right)}^{x_{n+1}\left(t\right)}\phi \left(x,t\right)\;\;d\;\;x\;,$$

bearing some success in capturing the transport contribution to the flux of energy. Unfortunately, it is not a flux variable. We shall argue that by dividing this quantity by $x_{n+1}\left(t\right)-x_{n}\left(t\right)$, either instantaneously or on average, one may reconcile the two views, to a certain degree. We shall also consider novel models of h(x,t) in the process.

## 2. Definition of the Flux—An Overview

We first outline some basic notation which we are going to use in this work. To avoid possible confusion, we shall refer to quantities indicating the flux of energy with variations of the greek letter ϕ. Thus, ϕ(x,t) will be indicating variations of the so-called continuous version of the flux, meaning that *x* is defined on an interval over the real line. Correspondingly, $\phi_{n}\left(t\right)$, or variations thereof, shall indicate discrete definitions, where *n* is in a subset of N. Quantities with the dimensions of an integrated flux are sometimes also called fluxes. Here, such integral quantities, not to be confused with heat fluxes, are denoted by the letter *j*. One can envision the existence of both j(x,t) continuous variations and $j_{n}\left(t\right)$ discrete variations. We shall adopt the mentioned choices of letters, despite the fact that often in the literature the letter *j* is used for both fluxes and integrated fluxes. We elect to do so also on the ground of dimensional considerations.

Correspondingly, h(x,t) is an energy density variable. For energy variables, we instead use the letters *e* or ϵ. For example, $e_{n}\left(t\right)$ may denote a specific version of the discrete energy associated with the *n*-th particle. Traditionally, h(x,t) is a sum of Dirac’s delta functions located at the particles positions. Instead, we shall cover different definitions (better called “models”) of h(x,t), to each one of which a different set of mathematical relations can be derived for the fluxes.

We consider a one-dimensional chain of *N* moving particles interacting classically via nearest-neighbor interaction. For simplicity, we shall assume that all masses are equal and unitary:

(9) $$H=\sum_{n=1}^{N}\frac{{\dot{x}}_{n}^{2}}{2}+\sum_{n=0}^{N}V\left(x_{n+1}-x_{n}\right)\;,$$

where $x_{0}=0$ and $x_{N+1}=a\left(N+1\right)=L$. Preferably, we shall assume that V(x) has a global minimun in x=a, which is also the lattice spacing. The dynamics is assumed to follow Newtonian dynamics, with the additional supplementation of heat baths at the left and right boundaries, which can generate a temperature gradient. This means that $x_{1}$ and $x_{N}$ are not merely Newtonian. We shall call “bulk” particles (n=2,…,N−1) those that are subject solely to Newtonian interactions with their nearest-neighbors.

We shall further assume that the order of the particles is conserved and within the walls, i.e., $x_{0}<x_{1}<x_{2}<\ldots <x_{N}<x_{N+1}$ at all times. For example, V(x) may be of the Lennard–Jones type or, more generally, diverging positively at the origin. This excludes the traditional FPUT models from our treatment. However, it may be possible to generalize at least some of our results also to that case.

Following from the authors of [33,34], in stationary nonequilibrium states (NESS), we assume the well-posedness of the following problem, in the form of the continuity equation

(10) $$\frac{\partial h(x,t)}{\partial t}+\frac{\partial \phi (x,t)}{\partial x}=0\;,$$

where h(x,t) and ϕ(x,t) are, respectively, the energy density and the energy flux in the position *x* at time *t*. The problem now is to relate Equation (10) to the variables which are usually traced in computer simulations, like positions and momenta of all the particles. In doing so, one would want to translate Equation (10) into an analogous expression for the local dynamical variables, adopting a suitable definition of energy flux in function of positions and momenta. This definition should (at least) guarantee the constancy of the average energy flux along the chain in NESS. Further, it should properly identify the heat flux, which would be observed in a real-world experiment. We stress that, in principle, such well-behaved local definition, in terms of the particles’ velocities and momenta, could involve several particles at a time; that is, the exact form of the local heat flux in the vicinity of the average position of the *n*-th particles could involve not only the *n*-th particle and its nearest-neighbors, but also several other more distant neighbors. The hope is that Equation (10) can be translated into a definition of heat flux which is both local and locally defined (i.e., involving only nearest-neighbors).

Thus, Equation (10) must be transformed ad hoc in terms of the energy and energy flux variables $e_{n}\left(t\right)$ and $\phi_{n}\left(t\right)$, for each particle in the bulk. In [33], one defines

(11) $$h\left(x,t\right)=\sum_{n}e_{n}\left(t\right)\;\;\delta \left(x-x_{n}\left(t\right)\right)\;.$$

For us, the bare summation symbol must be intended to refer to the particles in the bulk. In what follows, we shall often omit the explicit dependence on time. Thus, the energy density is defined in the distributions’ space, and it is singularly concentrated in the positions occupied by the particles. There is some degree of arbitrariness in defining $e_{n}$ locally, as the only constraint is that the integral of h(x,t) between the chain’s extrema be the total energy. We shall illustrate a small sample of alternative possibilities; however, the authors of [33] adopt the following,

(12) $$e_{n}=\frac{{\dot{x}}_{n}^{2}}{2}+\frac{1}{2}\left[V\left(x_{n+1}-x_{n}\right)+V\left(x_{n}-x_{n-1}\right)\right]\;.$$

Two routes now follow. One is to disregard Equation (10) and just notice that ${\dot{e}}_{n}$ (the time derivative of $e_{n}$) can be written as a difference equation of the type (the dot stands for time derivative),

(13) $${\dot{e}}_{n}=-\left(\phi_{n}-\phi_{n-1}\right)\;,$$

where $\phi_{n}$ is,

(14) $$\phi_{n}=-\frac{1}{2}\left({\dot{x}}_{n+1}+{\dot{x}}_{n}\right)V^{\prime }\left(x_{n+1}-x_{n}\right)=\frac{1}{2}\left({\dot{x}}_{n+1}+{\dot{x}}_{n}\right)F\left(x_{n+1}-x_{n}\right)\;,$$

with $V^{\prime }\left(x\right)$ the derivative of *V* with respect to its argument and the force given by $F\left(x\right)=-V^{\prime }\left(x\right)$.

According to the authors of [33], Equation (13) suggests an alternative to the continuity Equation (10), in the form of a finite difference equation, i.e.,

(15) $${\dot{e}}_{n}=-\frac{j_{n}-j_{n-1}}{a},\;\;j_{n}=a\phi_{n}\;.$$

The grounds for this identification lie in the observation that *a* is approximately the average inter-particle distance, and therefore if the fluctuations are small, then $\frac{j_{n}-j_{n-1}}{a}$ is approximately the local gradient of the flux (since *a* is the range over which the $j_{n}$’s vary). In a sense, here *a* could be read as the microscopic unit of length for energy variations. The derivation of Equation (15) is so straightforward that $j_{n}$, there defined (or rather $\phi_{n}$), has been often used as the definition of heat flux in one-dimensional models [14,17,21,22]. Variations occur, either with *a* is taken as unitary, or using slightly different definitions [30,31]. There are however only marginal differences, which vanish when long time averages in NESS systems are considered.

Notice that here the dimensional misalignment of $j_{n}$ and $\phi_{n}$ is not a severe problem, as they only differ by a constant.

The second route suggested in [33] is to complement Equation (11) with the following identity,

(16) $$\phi \left(x,t\right)=\sum_{n}j_{n}\left(t\right)\;\;\delta \left(x-x_{n}\left(t\right)\right)\;,$$

and then to find the most appropriate definition of the $j_{n}$’s which guarantee the validity of Equation (10). This passage is the main interest of this work. In fact, there is no known rigorous mathematical scheme that accomplishes this program to the letter. We shall also notice that, formally, Equation (16) is incomplete, as the continuity equation implies an additional missing piece-wise term, as already noted in [34]. One is then content with resolving it (at least) in some approximated form.

The strategies adopted in [32,33,34] here depart. Notably, they arrive at somewhat different conclusions: in the analysis of [32,33], Equation (15) can be recovered as a low (average) temperature limit of the full general problem, which in turn is solved only in a small wave-number approximation. In the analysis in [34], using properties of stationary states, it is proved (irrespective of the underlying temperature range) that summing and averaging Equation (15) over the *n*’s gives a total energy flux between the two ends of the chain, exactly as it is expected assuming Equations (10), (11), and (16). As we shall see, it is not obvious that the two findings are consistent, as locally the two ensuing definitions do not coincide, even net of stationary states averages. On the other hand, the approach of [33] of focusing on $j_{n}$ insetad of $\phi_{n}$, albeit dimensionally problematic, has the great merit of posing the problem of distinguishing between what we here call conductive and convective contributions.

One must stress that the fine objective is to identify the correct local definition of heat flux, i.e., not only one which is correct globally, but one that guarantees that its average is constant along the chain. An incorrect definition would manifest itself as an imbalance between left and right, eventually summing to zero.

Assuming low wave numbers are the leading contribution to the Fourier transform of Equation (10), the authors of [32,33] arrive at

(17) $$j_{n}=\frac{1}{2}\;\;\left(x_{n+1}-x_{n}\right)\;\;\left({\dot{x}}_{n+1}+{\dot{x}}_{n}\right)F\left(x_{n+1}-x_{n}\right)+{\dot{x}}_{n}\;\;e_{n}$$

and observe that Equation (15) is recovered if $x_{n+1}-x_{n}≃a$ and ${\dot{x}}_{n}\;\;e_{n}≃0$. This is interpreted as showing that in the small-oscillations limit, i.e., a crystalline-like state, Equation (15) is a good approximation of the more generally valid Equation (17).

We notice that Equation (17) can be recast as

(18) $$j_{n}=\left(x_{n+1}-x_{n}\right)\;\;\phi_{n}+{\dot{x}}_{n}\;\;e_{n}\;.$$

As in a NESS $\langle \;{\dot{x}}_{n}\;\;e_{n}\;\rangle =-\langle \;x_{n}\;\;{\dot{e}}_{n}\;\rangle$, where 〈z〉 denotes the time average of z(t), in NESSs we further have

(19) $$\begin{array}{ccc} \langle \;j_{n}\;\rangle & = & \langle \;\left(x_{n+1}-x_{n}\right)\;\;\phi_{n}\;\rangle -\langle \;x_{n}\;\;{\dot{e}}_{n}\;\rangle =\;\langle \;\left(x_{n+1}-x_{n}\right)\;\;\phi_{n}\;\rangle +\langle \;x_{n}\left(\phi_{n}-\phi_{n-1}\right)\;\rangle \\ & = & \langle \;x_{n+1}\phi_{n}\;\rangle -\langle \;x_{n}\phi_{n-1}\;\rangle =\psi_{n}-\psi_{n-1}\;;\;\;\psi_{n}:=\langle \;x_{n+1}\phi_{n}\;\rangle \;. \end{array}$$

For $\langle \;j_{n}\;\rangle$ to conserve its sign with *n* in the bulk, $\psi_{n}$ cannot be alternating in sign with *n*. For the energy flux to be constant in the bulk, $\langle \;j_{n}\;\rangle =j$, and therefore $\psi_{n}=\psi_{n-1}+j$. In retrospect, the authors of [33] distinguish a low-energy regime, where $|\psi_{n-1}-\langle \;x_{n}\phi_{n}\;\rangle |$ can be neglected compared to $|\psi_{n}-\langle \;x_{n}\phi_{n}\;\rangle |$, and a high-energy regime where it cannot [37].

Dhar [34] proposes a slightly different analysis. After deriving the discrete definition (15), in substitution of the continuum flux definition (16), he proceeds to define his continuum version of the same quantity,

(20) $$\phi \left(x,t\right)=\sum_{n}j_{n}^{*}\left(t\right)\;\;\delta \left(x-x_{n}\left(t\right)\right)+\sum_{n}{}^{\prime }\Delta_{n}^{\phi }\left(t\right)\;\;\theta \left(x-x_{n}\left(t\right)\right)\;,$$

which satisfies,

(21) $$\frac{\partial h(x,t)}{\partial t}+\frac{\partial \phi (x,t)}{\partial x}=\phi_{1,L}\;\delta \left(x\right)+\phi_{N,R}\delta \left(x-L\right)\;,$$

where,

(22) $$j_{n}^{*}=e_{n}{\dot{x}}_{n}\;,$$

(23) $$\Delta_{n}^{\phi }=\phi_{n}-\phi_{n-1}\;,$$

h(x,t) is defined in Equations (11) and (12), the sum $\sum^{\prime }$ extends from 2 to N−2 (rather than from 2 to N−1), θ(x) is the Heavyside step function, and $\phi_{1,L}$ and $\phi_{N,R}$ are left and right boundary terms, respectively.

It is implicit in [34] how definition (20) leads to (21). Our purpose here is to formalize this argument, and we shall use several different models of the continuum energy density h(x,t) in the process. A crucial point of the analysis in [34] is that its author integrates ϕ(x,t) from 0 to *L* (the fixed boundary) to get the total current, and then performs NESS averages. Aided by several cancellations of bulk contributions and the choice of Maxwell baths, he, thus, shows that $\langle \;\phi \left(x,t\right)\;\rangle =\langle \;\phi_{n}\;\rangle$, with $\phi_{n}$ defined in (15), which in NESS’s must satisfy $\langle \;\phi_{n}\rangle =\langle \;\phi_{n-1}\;\rangle$.

In the following analysis, we shall consider only bulk terms, as we are interested in understanding the significance of the local definition of flux, to discriminate between the definitions (15) and (17). In fact, when considering the Equation (10), the work [33] privileges the quantity defined in Equation (17), of which Equation (15) is presented as a low-temperature approximation, yet it relies on a small wave-number approximation. The work [34] also relies on Equation (21), but it purports to assert that in NESS’s Equation (15) is consistent with it. In this case, only the total average flux is calculated, which makes it difficult to understand where the paradox comes from. To avoid confusion about the different treatment of boundary terms, we shall consider x∈(0,L), so that Equation (10) applies ∀x.

## 3. The Local Energy Flux Redefining the Energy Density

Given the distributions f(x,t) and g(x,t), we define the differential operator

(24) $$D\;\left(f,g\right)=\frac{\partial f}{\partial t}+\frac{\partial g}{\partial x}\;.$$

Given u(t) and w(t), we also define the integral operator

(25) $$I_{u,w}\;f=\int_{u(t)}^{w(t)}f\left(x,t\right)\;dx\;.$$

We now replace the definition of the energy density of Equation (11) with a new one. At this stage, this choice of model should not be regarded as favorable with respect to that of Equation (11) nor to others which will follow in the next sections. However, it is suitable to highlight some of the intricacies of such formalizations. We shall derive expressions for the energy flux very similar to those contained in [33], provided some key steps are used in the derivations.

Thus, we posit that for 0<x<L, h(x,t) and ϕ(x,t) satisfy

(26) D(h,ϕ)=0,

and that h(x,t) further satisfies (with $x_{n}=x_{n}\left(t\right)$ intended ∀n)

(27) $$h\left(x,t\right)=\sum_{n}K_{n}\;\delta \left(x-x_{n}\right)+\sum_{n}{}^{\prime }\frac{V_{n}}{x_{n+1}-x_{n}}\left[\theta \left(x-x_{n}\right)-\theta \left(x-x_{n+1}\right)\right]\;,$$

where θ(x) is the Heavyside step function and the following hold,

(28) $$K_{n}=\frac{{\dot{x}}_{n}^{2}}{2}\;;\;\;V_{n}=V\left(x_{n+1}-x_{n}\right)\;.$$

The rationale for assuming the form (27) is the following. The kinetic energy is entirely concentrated at the particles’ positions, it is therefore delta-distributed. The potential energy contributed by the particles *n* and n+1, instead, is taken as being due to $V_{n}$, but it is spread uniformly over the distance $x_{n+1}-x_{n}$. In fact, the term in square parentheses in Equation (27) is 1 if $x_{n}<x<x_{n+1}$ and zero otherwise. The division by $x_{n+1}-x_{n}$ guarantees the uniform distribution of the potential energy in that interval. We call the model definition of Equation (27) “hybrid”, to distinguish it from that of Equation (11), which is solely delta-distributed, and one which will follow later in this work, which is solely piecewise-distributed.

We notice that both works [33,34] use the definition (11) rather than (27): we shall come back at a later stage on that definition. We do not claim that the definition (27) is different from (11) in a fundamental sense. However, we point out that the definition (27) has the formal advantage that both h(x,t) and ϕ(x,t) turn out to comprise both delta-distributed and stepwise-distributed terms. Conversely, from the solely delta-distributed definition (11) of h(x,t), a delta- and stepwise-distributed ϕ(x,t) follows.

After defining the shorthand for the force $F_{n}=F\left(x_{n+1}-x_{n}\right)$, via the equations of motion, we shall make extensive use of the identities,

(29) $${\dot{K}}_{n}={\dot{x}}_{n}\left(F_{n-1}-F_{n}\right)\;;\;\;{\dot{V}}_{n}=\left({\dot{x}}_{n}-{\dot{x}}_{n+1}\right)\;F_{n}\;.$$

We also denote

(30) $$X_{n}=x_{n+1}-x_{n}\;;\;\;\;{\dot{X}}_{n}={\dot{x}}_{n+1}-{\dot{x}}_{n}\;;$$

(31) $$R_{n}=\theta \left(x-x_{n}\right)-\theta \left(x-x_{n+1}\right)\;.$$

$R_{n}$ is the rectangular function which is unity in the interval $x\in (x_{n},x_{n+1})$, eventually 1/2 at the two boundaries, and zero elsewhere. We shall use the identities,

(32) $$\partial_{x}\theta \left(x\right)=\delta \left(x\right)\;;\;\;\;\partial_{x}\left(x\;\theta (x)\right)=\theta \left(x\right)\;;$$

(33) $$\partial_{t}\;\delta \left(x-x_{n}\right)=-{\dot{x}}_{n}\;\partial_{x}\;\delta \left(x-x_{n}\right)\;,$$

where $\partial_{t}$ and $\partial_{x}$ denote time and coordinate derivative, respectively, with the latter also standing for derivative with respect to the function’s argument.

Therefore the definition (27) can be rewritten:

(34) $$h\left(x,t\right)=\sum_{n}K_{n}\;\delta \left(x-x_{n}\right)+\sum_{n}{}^{\prime }\frac{V_{n}\;R_{n}}{X_{n}}\;,$$

and, with a suitable choice of ϕ(x,t), we wish to write its time derivative in the form,

(35) $$\partial_{t}h\left(x,t\right)=-\partial_{x}\;\phi \left(x,t\right)\;,$$

which can be thus constructed:

(36) $$\begin{array}{c} \partial_{t}h\left(x,t\right)=\sum_{n}\left({\dot{K}}_{n}\;\delta \left(x-x_{n}\right)-K_{n}{\dot{x}}_{n}\;\partial_{x}\;\delta \left(x-x_{n}\right)\right)\;\;\;\;\;\\ \;\;\;+\sum_{n}{}^{\prime }(\frac{{\dot{V}}_{n}\;R_{n}}{X_{n}}-\frac{V_{n}{\dot{X}}_{n}\;R_{n}}{X_{n}^{\;2}}+\frac{V_{n}}{X_{n}}\left({\dot{x}}_{n+1}\delta \left(x-x_{n+1}\right)-{\dot{x}}_{n}\delta \left(x-x_{n}\right)\right)\;, \end{array}$$

By using the identities (32), then noticing that

(37) $$R_{n}=\partial_{x}\left[\left(x-x_{n}\right)\;\theta \left(x-x_{n}\right)-\left(x-x_{n+1}\right)\;\theta \left(x-x_{n+1}\right)\right]$$

and using

(38) $$\sum_{n}{\dot{K}}_{n}\theta \left(x-x_{n}\right)=\sum_{n}{}^{\prime }F_{n}\left({\dot{x}}_{n+1}\theta \left(x-x_{n+1}\right)-{\dot{x}}_{n}\theta \left(x-x_{n}\right)\right)\;,$$

after some lengthy algebra we arrive at

(39) $$\phi \left(x,t\right)=\sum_{n}K_{n}{\dot{x}}_{n}\;\delta \left(x-x_{n}\right)+\sum_{n}{}^{\prime }\left(F_{n}+\frac{V_{n}}{X_{n}}\right)\left(x\;{\dot{X}}_{n}+x_{n+1}{\dot{x}}_{n}-x_{n}{\dot{x}}_{n+1}\right)\frac{R_{n}}{X_{n}}\;.$$

Therefore, Equation (39) defines the flux ϕ(x,t) associated with the bulk energy density of Equation (34), where *x* lies in the open set x∈(0,L). In principle, one wants to calculate the flux within a finite region of fixed boundaries *b* and *c*, thus obtaining a properly defined heat flux. Unfortunately, the task of using Equation (39) to this aim is unlikely to be practical, as countably many terms contribute to the energy flux in that region. Even by choosing $b=\langle x_{n}\rangle$ and $c=\langle x_{n+1}\rangle$, although the *n*-th and (n+1)-th particles are certainly the leading contributors, due to the large fluctuations, one cannot discount the contributions to the flux of the nearby particles, occasionally transversing the region [b,c]. On the other hand, solving the equations of motion to determine ϕ(x,t) would be prohibitive.

Equation (39) differs from Equation (20) in one interesting aspect. Dhar [34] comments on Equation (20), revealing that it naturally distinguishes transport terms, the delta-type contributions, from interaction terms, the piecewise contributions. Conversely, in Equation (39), only the kinetic terms are of the “transport” type, when the locution is adopted in the same sense. As we shall point out, the effect of applying the operator $I_{x_{n},x_{n+1}}$ to ϕ will be to reconcile the two interpretations.

We now wish to integrate Equation (39), to pass from an energy flux to some form of integrated energy flux, given in explicit terms. We shall use the operator $I_{x_{n},x_{n+1}}$ to show that this is precisely the action that one needs to take to recover expressions echoing that of Equation (17); the fact that the integral operator thus defined serves this objective is one of the main observations of this work.

As $x_{n}$ and $x_{n+1}$ evolve in time, we do not expect this operation to return a heat flux in the conventional sense. Instead, for every *t*, it is the instantaneous energy flux integrated between the positions occupied respectively by the particles labeled *n* and n+1. If one wishes, it can be viewed as a putative integrated heat flux in a reference frame which expands and contracts together with the length $x_{n+1}-x_{n}$. Some reason for consideration for this quantity can be drawn from the observation that the particles’ positions and velocities are the typical variables monitored in computer simulations. In this specific section, such integration is carried out once the definitions (10) and (34) are assumed.

In what follows, delta functions will be considered within the theory of test functions, in such a way that $\int_{0}^{\infty }\delta \left(x\right)f\left(x\right)dx=f\left(0\right)/2$ is assumed. Then, for pairs of particles in the bulk, Equation (39) yields

(40) $$\begin{array}{c} I_{x_{n},x_{n+1}}\phi =\int_{x_{n}}^{x_{n+1}}\phi \left(x,t\right)\;\;dx=\frac{1}{2}\;\;\left(x_{n+1}-x_{n}\right)\;\;\left({\dot{x}}_{n+1}+{\dot{x}}_{n}\right)F\left(x_{n+1}-x_{n}\right)\;\\ +\;\frac{1}{2}\;\;{\dot{x}}_{n}\;\;K_{n}+\frac{1}{2}\;\;{\dot{x}}_{n+1}\;\;k_{n+1}+\frac{1}{2}\;\;\left({\dot{x}}_{n}+{\dot{x}}_{n+1}\right)\;\;V_{n}=\;\\ =\left(x_{n+1}-x_{n}\right)\;\;\phi_{n}+\;\frac{1}{2}\;\;{\dot{x}}_{n}\;\;K_{n}+\frac{1}{2}\;\;{\dot{x}}_{n+1}\;\;k_{n+1}+\frac{1}{2}\;\;\left({\dot{x}}_{n}+{\dot{x}}_{n+1}\right)\;\;V_{n}\;.\; \end{array}$$

This expression bears a striking resemblance with that of $j_{n}$ defined in Equations (17) and (18). Notice that of the two terms in ${\dot{x}}_{n}\;\;e_{n}={\dot{x}}_{n}\;\;K_{n}+{\dot{x}}_{n}\;\;V_{n}$, in the r.h.s. of Equations (17) and (18), here ${\dot{x}}_{n}\;\;K_{n}$ is replaced by a mean kinetic energy contribution by the two particles, $\frac{1}{2}\;\;{\dot{x}}_{n}\;\;K_{n}+\frac{1}{2}\;\;{\dot{x}}_{n+1}\;\;k_{n+1}$. Concurrently, ${\dot{x}}_{n}\;\;V_{n}$ is replaced by a ‘center of mass’ type term, $\frac{1}{2}\;\;\left({\dot{x}}_{n}+{\dot{x}}_{n+1}\right)\;\;V_{n}$. Equation (40) comprises a conductive (interaction) and a convective (trasnport) term, the former of which is traditionally identified with $\left(x_{n+1}-x_{n}\right)\;\;\phi_{n}$, rather than barely $\phi_{n}$, as given in Equation (15). Loosely speaking, although different, Equation (40) is thus in the same class of Equation (18), as opposed to Equation (15). This is an indication that also a definition such as that of Equation (18), first derived just as an approximation in the long wavelength limit, might be recast in some exact form, involving the integration of ϕ(x,t) over the moving extremes. We shall come back to such suggestion later in this article, showing the effect of slightly changing the definition of h(x,t). It must be observed that the hypothesis of conserved positional order of the particles, for example due to infinite or diverging contact repulsion, is central to this consideration. FPUT models without repulsion are more difficult to treat within our present formalism and analysis.

We now show that Equation (40) has a certain extent of atypical, if not yet pathological character. First, by construction, we have that, once the definitions (34) and (39) for *h* and ϕ are, respectively, assumed, then

(41) $$I_{x_{n},x_{n+1}}D\;\left(h,\phi \right)=\int_{x_{n}}^{x_{n+1}}\left(\frac{\partial h}{\partial t}+\frac{\partial \phi }{\partial x}\right)\;\;dx=0\;,$$

as the integrand is null.

The point about the definition (40) is that it does not apparently satisfy any continuity equation, not even in the form of a difference equation. Indeed, it is an integrated flux. This is an additional concern, and not strictly tied to the improper definition of the flux via time-dependent boundaries of the domain of integration (the $x_{n}\left(t\right)$ and $x_{n+1}\left(t\right)$). It is of crucial relevance that we are facing a difficult problem, which is to come up with a definition of heat flux at the scales of molecular interactions, i.e., where the domain of integration is microscopic. Such concerns about equations such as (18) or (40) are only resolvable when one considers fixed boundaries at distances much larger than the smaller macroscopic distance (in fact in [34] one appreciates that at the scale of *L*, the definition is correct).

To show that the consideration of the operator $I_{x_{n},x_{n+1}}$ does have some merit in our context, let us appreciate its effect on h(x,t) defined in Equation (34):

(42) $$I_{x_{n},x_{n+1}}h=\int_{x_{n}}^{x_{n+1}}h\left(x,t\right)\;dx=\frac{1}{2}\;\;K_{n}+\frac{1}{2}\;\;K_{n+1}+V_{n}\;,$$

therefore

(43) $$\frac{d}{dt}\int_{x_{n}}^{x_{n+1}}h\left(x,t\right)\;dx=\frac{1}{2}\;\;{\dot{K}}_{n}+\frac{1}{2}\;\;{\dot{K}}_{n+1}+{\dot{V}}_{n}=-\left(\phi_{n}^{†}-\phi_{n-1}^{†}\right)\;,$$

(44) $$\phi_{n}^{†}=\frac{1}{2}\;\;{\dot{x}}_{n+1}\;\;\left(F_{n+1}+F_{n}\right)\;.$$

In a stationary state, from Equations (29) and the condition $\langle \dot{K}\rangle =\langle \dot{V}\rangle =0$, one can easily verify that

(45) $$\langle \phi_{n}^{†}\rangle =\langle \phi_{n}\rangle \;,$$

therefore the definitions of the fluxes ϕ or $\phi^{†}$ are compatible in stationary regimes, the former coming from definitions (11) and (12), the latter from (34).

Equation (43) is an interpretation of the continuity equation: it is the time derivative of the integral of the energy density (34), over a prescribed domain, written as the negative difference of energy flux terms at the boundary. As we shall see, one can conjure up a plethora of models of h(x,t) with the possibility of such an interpretation, only a reduced sample of which will be presented in the next sections. In the case of the present model (40), however, there is still an non-fitting aspect, which is that the equality

(46) $$\frac{d}{dt}\int_{x_{n}}^{x_{n+1}}h\left(x,t\right)\;dx=\int_{x_{n}}^{x_{n+1}}\frac{\partial }{\partial t}h\left(x,t\right)\;dx$$

cannot be formally established, in spite of the validity of the identity (41). In fact, the latter entails the mutual cancellation of irreducibly singular terms, which arise from the terms $K_{n}{\dot{x}}_{n}\;\partial_{x}\;\delta \left(x-x_{n}\right)$ in Equation (36) when evaluated at the boundaries of integration. A simpler, more intuitive explanation of why Equation (46) does not hold here has been anticipated in the Introduction. By Leibniz’s rule, the two terms on the right and left sides of Equation (46) differ by contributions involving the evaluation of h(x,t) at its singular points, the $x_{n}$’s. Thus, by Equation (43), $\phi_{n}^{†}$ is a useful flux variable, but in general $\phi_{n}^{†}\left(t\right)\neq \phi \left(x_{n}\left(t\right),t\right)$.

However, this obstacle is formally circumventable by either of two means: by changing ad hoc the domain of integration, or the definitions of the energy density h(x,t). In each instance, we obtain slightly different definitions of the fluxes, in some of the cases compatible with each other in stationary states.

## 4. Local Energy Flux: Changing the Domain of Integration

Definition (34) is not especially suitable for further analysis, as the ensuing formulas are somewhat cumbersome and not straightforwardly interpretable. For now, it is much more straightforward to turn to the traditional definition of Equation (11), by time differentiating

(47) $$\begin{array}{c} \partial_{t}h\left(x,t\right)=\sum_{n}\left({\dot{e}}_{n}\;\delta \left(x-x_{n}\right)-e_{n}{\dot{x}}_{n}\;\partial_{x}\;\delta \left(x-x_{n}\right)\right)\;\;\;\;\\ \;\;\;=\sum_{n}\left(\left(\phi_{n+1}-\phi_{n}\right)\;\delta \left(x-x_{n}\right)-e_{n}{\dot{x}}_{n}\;\partial_{x}\;\delta \left(x-x_{n}\right)\right)\;\;\\ \;\;\;=-\sum_{n}\left(\phi_{n}\;\left(\delta \left(x-x_{n}\right)-\delta \left(x-x_{n+1}\right)\right)+e_{n}{\dot{x}}_{n}\;\partial_{x}\;\delta \left(x-x_{n}\right)\right)\;. \end{array}$$

Thus, in this case,

(48) $$\begin{array}{c} \phi \left(x,t\right)=\sum_{n}{\dot{x}}_{n}e_{n}\;\delta \left(x-x_{n}\right)+\sum_{n}{}^{\prime }\phi_{n}\;\;R_{n}\;. \end{array}$$

If, again, we apply the integral operator $I_{x_{n},x_{n+1}}\phi$ to this definition of flux, we now get

(49) $$\begin{array}{cc} I_{x_{n},x_{n+1}}\phi =\int_{x_{n}}^{x_{n+1}}\phi \left(x,t\right)\;\;dx=\left(x_{n+1}-x_{n}\right)\;\;\phi_{n}+\;\frac{1}{2}\;\;{\dot{x}}_{n}\;\;e_{n}+\frac{1}{2}\;\;{\dot{x}}_{n+1}\;\;e_{n+1}\;.\; & \end{array}$$

This is also similar to Equation (18), and to some extent to Equation (40). Compared to Equation (18), it is more symmetric, as both contributions to the integrated flux between the particles *n* and n+1 are expected to bear a contribution from both particles, not only limited to $\left(x_{n+1}-x_{n}\right)\;\;\phi_{n}$.

Similarly to the case of Equation (40), we could not find an obvious interpretation for this total energy flux locally defined that we could accommodate in the form of a difference equation. Indeed, this may echo the fact that it is an integral quantity. On the other hand, h(x,t) and ϕ(x,t) defined in Equations (11) and (48) still satisfy Equation (41), by definition. Concurrently, the equality (46) cannot be established here too, because of the singularity brought about at integration by the last term in Equation (47). Nevertheless,

(50) $$I_{x_{n},x_{n+1}}h=\int_{x_{n}}^{x_{n+1}}h\left(x,t\right)\;dx=\frac{1}{2}\;\;K_{n}+\frac{1}{2}\;\;K_{n+1}+\frac{1}{4}\;\;\left(V_{n-1}+2\;\;V_{n}+V_{n+1}\right)$$

and

(51) $$\frac{d}{dt}\int_{x_{n}}^{x_{n+1}}h\left(x,t\right)\;dx=-\frac{1}{2}\;\;\left(\phi_{n+1}-\phi_{n-1}\right)=-\frac{1}{2}\;\;\left(\left(\phi_{n+1}+\phi_{n}\right)-\left(\phi_{n}+\phi_{n-1}\right)\right)\;.$$

Again, the steady-state averages are consistent with previous definitions.

To circumvent the ambiguity of resting on the convention $\int_{0}^{\infty }\delta \left(x\right)f\left(x\right)dx=f\left(0\right)/2$, which we have extensively employed so far, and to test the possibility that the equality (46) holds, for ad hoc operators, we introduce

(52) $$y_{n}=\frac{1}{2}\;\left(x_{n}+x_{n+1}\right)\;,$$

which is the mid-point (or centre of mass variable) between neighbouring particles. Thus, one immediately has

(53) $$I_{y_{n-1},y_{n}}h=\int_{y_{n-1}}^{y_{n}}h\left(x,t\right)\;dx=K_{n}+\frac{1}{2}\;\;\left(V_{n-1}+V_{n}\right)=e_{n}\;.$$

Then, by noticing that $\int_{-a}^{a}\partial_{x}\delta \left(x\right)dx=0$ for a>0, from Equation (47) follows that

(54) $$\frac{d}{dt}\int_{y_{n-1}}^{y_{n}}h\left(x,t\right)\;dx=\int_{y_{n-1}}^{y_{n}}\frac{\partial h(x,t)}{\partial t}\;dx=-\left(\phi_{n}-\phi_{n-1}\right)\;,$$

which, of course, also implies

(55) $$\int_{y_{n-1}}^{y_{n}}\frac{\partial \phi (x,t)}{\partial x}\;dx=\phi_{n}-\phi_{n-1}\;.$$

Therefore, the flux variables $\phi_{n}$ act essentially as effective primitives of the local flux derivative $\partial_{x}\phi \left(x,t\right)$, under this special integral operator, and thus as legitimate finite flux measures. Indeed, by Leibniz’s integral rule, since the energy density h(x,t) is zero at the $y_{n}$’s, then $\phi_{n}\left(t\right)=\phi \left(y_{n}\left(t\right),t\right)$. They measure the out- and in-flux to the right and left of the *n*-th particle, at any given time. The importance of the first equality in Equation (54) resides in the fact that it usually holds for fixed boundaries of integration, but here it holds despite the boundaries are moving. We stress once again the fact that the well ordering of the particles positions, for example due to diverging short-range repulsion, is an essential ingredient in the construction. Concurrently, by using the $y_{n}$’s as extremes of integration, one is excluding the contribution of transport across the boundaries.

For completeness, from Equation (48) we derive,

(56) $$\begin{array}{cc} I_{y_{n-1},y_{n}}\phi =\int_{y_{n-1}}^{y_{n}}\phi \left(x,t\right)\;\;dx=\frac{1}{2}\;\;\left(x_{n}-x_{n-1}\right)\;\;\phi_{n-1}+\frac{1}{2}\;\;\left(x_{n+1}-x_{n}\right)\;\;\phi_{n}+{\dot{x}}_{n}\;\;e_{n}\;.\; & \end{array}$$

This is the closest derived expression to the definition (18) so far. The first two terms account for the fact that we are considering the region to the left and right of the *n*-particle, rather than a pure inter-particle interval, and thus we obtain a weighted average of the usual contribution $\left(x_{n+1}-x_{n}\right)\;\;\phi_{n}$.

## 5. Local Energy Flux: Shifting the Location of the Energy

In this section, we show the effect of altering the definition of the points where the energy must be considered located. It is chiefly a formal exercise, which however should further clarify the complexity of handling quantities at the microscopic scales. Let us now define the energy to be singularly concentrated at the mid points between particles, i.e.,

(57) $$h\left(x,t\right)=\sum_{n}\epsilon_{n}\left(t\right)\;\;\delta \left(x-y_{n}\left(t\right)\right)\;.$$

(58) $$\epsilon_{n}=\frac{1}{2}\;\;K_{n}+\frac{1}{2}\;\;K_{n+1}+V_{n}$$

with $y_{n}$ defined in Equation (52). The rationale is that the energy of competence of $y_{n}$, which is the mid point between $x_{n}$ and $x_{n+1}$, is the total potential energy contributed by the two particles, plus half of the kinetic energy of each. Clearly,

(59) $$I_{x_{n},x_{n+1}}h=\int_{x_{n}}^{x_{n+1}}h\left(x,t\right)\;dx=\epsilon_{n}$$

(60) $$\frac{d}{dt}\int_{x_{n}}^{x_{n+1}}h\left(x,t\right)\;dx=\int_{x_{n}}^{x_{n+1}}\frac{\partial h(x,t)}{\partial t}\;dx={\dot{\epsilon }}_{n}=-\left(\phi_{n}^{†}-\phi_{n-1}^{†}\right)$$

with $\phi_{n}^{†}$ consistent with $\phi_{n}$ in NESS, as we have discussed following Equation (43). It directly follows that

(61) $$\int_{x_{n}}^{x_{n+1}}\frac{\partial \phi (x,t)}{\partial x}\;dx=\phi_{n}^{†}-\phi_{n-1}^{†}$$

Further, one can verify that

(62) $$\begin{array}{cc} I_{x_{n},x_{n+1}}\phi =\int_{x_{n}}^{x_{n+1}}\phi \left(x,t\right)\;\;dx=\left(x_{n+1}-x_{n}\right)\;\;\phi_{n}^{†}+\frac{1}{2}\;\;\left({\dot{x}}_{n}+{\dot{x}}_{n+1}\right)\;\;\epsilon_{n}\; & \end{array}$$

The structure of Equation (62) is the same as that of Equations (18), (49), and (56). Yet, we could not find any of them to be consistent in NESS. On the contrary, either $\phi_{n}$ or $\phi_{n}^{†}$ differences appear in Equations (43), (51), (54), and (60), with the addition that the formal constructions that led to Equations (54) and (60) also satisfy Equation (46). Plus, the flux variables $\phi_{n}$ and $\phi_{n}^{†}$ have equal averages in NESS, and they represent solid definitions with respect to the various formal definitions of microscopic energy densities that we have so far tested.

For the present case, it is thus implied that $\phi_{n}\left(t\right)=\phi \left(x_{n}\left(t\right),t\right)$, because the $x_{n}$’s are not the points where the energy is concentrated. In such formalization, the crossing of barriers is thus a priori excluded from contributing to the energy flux. In the section following the next, we shall try to circumvent this limitation and derive its consequences.

We point out that several other variations of how to label and allocate the energy density can be conjured up. For example, say the potential energy is entirely allocated at the $y_{n}$’s and the kinetic energy at the $x_{n}$’s points. We do not pursue this further here, because it would become a scholastic exercise. In many variations, the results would be consistent with the previous considerations. Nevertheless, as just mentioned above, we shall propose one specific (less conventional) variation that may shed some more light on this issue.

Before doing that, in the next section we want to explore some of the past descriptions, to relate them more closely to our present analysis. We do this here, as later on we shall depart from them more conspicuously.

## 6. Relation to Other Approaches

In [32,33], Equations (12) and (16) define the energy density and local flux, respectively. Notice that both h(x,t) and ϕ(x,t) are presumed to be sums of delta functions. The two quantities are then Fourier transformed so that, in the short-wavenumber limit of the exponential bases, the Fourier transformed quantities are both purely imaginary, and the $j_{n}$’s rest defined by term-by-term correspondence, giving rise to Equation (17). As already pointed out in [34], if h(x,t) is the sum of delta functions, then ϕ(x,t) must contain both delta and step functions, as we have already reported in Section 2.

Starting from this position, we here show how to use the idea in [32,33] to arrive, via Fourier analysis, to conclusions similar to those of the preceding sections. Additionally, we may better discuss the short-wavenumber limit. Thus, assuming h(x,t) to be the sum of delta functions centered at the particles’ positions, we write

(63) $$\phi \left(x,t\right)=\sum_{n}j_{n}^{\delta }\left(t\right)\;\;\delta \left(x-x_{n}\left(t\right)\right)+\sum_{n}{}^{\prime }\phi_{n}^{\theta }\left(t\right)\;\;\theta \left(x-x_{n}\left(t\right)\right)$$

The choice of denoting the (yet unknown) coefficients of the sums $j_{n}^{\delta }$ and $\phi_{n}^{\theta }$ reflects their dimensionality, of an integrated flux and of a flux, respectively.

In Fourier space, assuming

$$\tilde{h}\left(k,t\right)=\int_{-\infty }^{\infty }h\left(x,t\right)e^{-ikx}\;\;dx\;\;\mathrm{and}\;\;\tilde{\phi }\left(k,t\right)=\int_{-\infty }^{\infty }\phi \left(x,t\right)e^{-ikx}\;\;dx\;,$$

we have

(64) $$\tilde{h}\left(k,t\right)=\sum_{n}e_{n}e^{-ikx_{n}};\;\;\tilde{\phi }\left(k,t\right)=\sum_{n}\left[j_{n}^{\delta }+\left(\frac{1}{ik}+\pi \delta \left(k\right)\right)\phi_{n}^{\theta }\right]e^{-ikx_{n}}\;.$$

For the continuity equation to hold:

(65) $$\frac{\partial \tilde{h}\left(k,t\right)}{\partial t}=-ik\tilde{\phi }\left(k,t\right)\;,$$

where

(66) $$\frac{\partial \tilde{h}\left(k,t\right)}{\partial t}=\sum_{n}\left({\dot{e}}_{n}-ik{\dot{x}}_{n}e_{n}\right)e^{-ikx_{n}}\;.$$

Using kδ(k)=0, Equations (64)–(66) can be combined, and they give

(67) $$\sum_{n}\left({\dot{e}}_{n}-ik{\dot{x}}_{n}e_{n}\right)e^{-ikx_{n}}=-\sum_{n}\left(\phi_{n}^{\theta }+ikj_{n}^{\delta }\right)e^{-ikx_{n}}\;,$$

or,

(68) $$\phi_{n}^{\theta }=-{\dot{e}}_{n}=\phi_{n}-\phi_{n-1}\;\;\mathrm{and}\;\;j_{n}^{\delta }={\dot{x}}_{n}e_{n}\;.$$

Therefore,

(69) $$\phi \left(x,t\right)=\sum_{n}{\dot{x}}_{n}e_{n}\;\;\delta \left(x-x_{n}\right)+\sum_{n}\left(\phi_{n}-\phi_{n-1}\right)\;\;\theta \left(x-x_{n}\right)\;,$$

which is easily rearranged to give exactly Equation (48). In [34], one may identify the first term as representing transport of matter, thus we say “convection”, whereas the second one represents particles’ interaction, thus we say “conduction;’.

We notice in the procedure outlined in [32,33], after using the expression (16) instead of (63), the exponential terms of $\dot{\tilde{h}}\left(k,t\right)$ in Equation (66) are expanded in *k*, which leads to an apparent *k* as lower order. Contrarily here, from Equation (67), which is exact assuming the expression (63), wee see a finite term as the zeroth order in *k*, i.e., the equality on the left in Equation (68). This further supports the idea that $\phi_{n}$ is a robust definition of energy flux, since it captures the infinite length limit. Put in other terms, if the system of particles in confined in the region (0,L), then $\tilde{h}\left(0,t\right)=\int_{0}^{L}h\left(x,t\right)\;\;dx$ is the total energy, and $\phi_{n}$ captures the energy flux of competence of such total energy. We stress that the choice of model for h(x,t) is functional to the conclusion.

Without resorting to the dangerous expansion of the exponential basis, suffice it to observe that, in addition to the $\phi_{n}$’s, for all non-zero wavelengths, one just adds $ik{\dot{x}}_{n}e_{n}$ to get the complete expression of the energy flux coefficients in Fourier space. Numerically, if one were to consider only *k* much smaller than 1/L and expand the exponential, then the partial terms in the sum of Equation (67) would be approximated by $≃\left(\phi_{n}-\phi_{n-1}\right)\left(1-ikx_{n}\right)+ik{\dot{x}}_{n}e_{n}$, which finally makes some justice of the proposition in [32,33].

Considering the case of NESS averages, one easily sees that

(70) $$\langle {\dot{e}}_{n}\;\;e^{-ikx_{n}}\rangle =ik\langle {\dot{x}}_{n}e_{n}\;\;e^{-ikx_{n}}\rangle$$

and, consequently,

(71) $$\langle \left(\phi_{n-1}-\phi_{n}\right)\;\;e^{-ikx_{n}}\rangle =ik\langle {\dot{x}}_{n}e_{n}\;\;e^{-ikx_{n}}\rangle \;.$$

On average, the terms in the summand of Equation (67) cancel each other out. This is another view on the balancing of the energy flux contributions in NESS, already pointed out in [34] via integration from 0 to *L*.

The structure of Equation (48) has already been discussed in [34], so we just propose a few additional comments. Fix *x* and evaluate the flux ϕ(x,t) through Equation (48). The $\phi_{n}$’s capture the interaction contribution to the flux and can be easily extracted from simulation data. For example, $\phi \left(x,t\right)=\phi_{n}$ if $x_{n}<x<x_{n+1}$, or $\phi \left(x,t\right)=\phi_{n-1}$ if $x_{n-1}<x<x_{n}$, and so on. Conversely, the ${\dot{x}}_{n}e_{n}$’s terms represent the contribution to the flux due to the transport of particles across a given barrier. Due to their singular nature, they cannot typically be monitored with the above procedure, since $x=x_{n}$ has probability almost surely zero to occur in the simulation. For strongly interacting (low temperature) systems, this poses a negligible problem, but not for weakly interacting particles (except at contact), as also pointed out in [33,37]. One may include the singular terms in the analysis, for example, by taking two slices at two fixed position, separated by a distance ϵ≪1, and estimate

(72) $$\int_{x}^{x+\epsilon }\phi \left(x,t\right)\;\;dx≃\phi \left(x,t\right)\epsilon \;.$$

The length ϵ should be small, but sufficiently large to permit the monitoring of the occurrences of $x<x_{n}<x+\epsilon$ (for any *n*). One sees “kicks” by the particles when they occasionally contribute to the estimation of the flux, during their passage inside the strips. However, in our analysis, outlined in the previous sections, we have seen that the expression for the transport terms depends on the chosen form for h(x,t), despite several such expressions are compatible with each other in many other respects.

The usefulness of the integrated flux quantity $j_{n}$ lies in the possibility to capture the contribution of the matter transport terms, ${\dot{x}}_{n}e_{n}$. In fact, once *b* and *c* are some fixed positions in space, the identity

(73) $$\frac{d}{dt}\int_{b}^{c}h\left(x,t\right)\;dx=\int_{b}^{c}\frac{\partial h(x,t)}{\partial t}\;dx=-\int_{b}^{c}\frac{\partial \phi (x,t)}{\partial x}\;dx=\phi \left(b,t\right)-\phi \left(c,t\right)$$

alone cannot be used in practice, to account for the transport of matter across either *b* or *c* (due to the singularity nature of the problem). Furthermore, we only obtain a measure of a difference of fluxes, not of either ϕ(b) or ϕ(c). On the contrary,

(74) $$\int_{b}^{c}\phi \left(x,t\right)\;\;dx$$

measures the cumulated flux between *b* and *c*. It may be reinterpreted as c−b times the average flux in the region b<x<c. As pointed out in [34], this is exactly true if b=0 and c=L, and all the particles from 1 to *N* are confined in that region. Then, in steady states, and assuming L=Na, $\langle \int_{0}^{L}\phi \left(x,t\right)\;\;dx\rangle =L\langle \phi_{n}\rangle =N\langle j_{n}\rangle$. Indeed, all transport of matter is confined to within the region (0,L). This observation also gives justice of the well-definedness of the integral operator $I_{y_{n-1},y_{n}}$ discussed in Section 4, as the interval of integration is never trespassed by the particles.

Note that $\langle \int_{0}^{L}\phi \left(x,t\right)\;\;dx\rangle =L\langle \phi_{n}\rangle$ (with $\langle \phi_{n}\rangle$ constant everywhere) does not guarantee, per se, that 〈ϕ(x,t)〉 is uniformly distributed in space. In general, $\langle x_{n+1}-x_{n}\rangle$ is not independent of *n*, e.g., it is inversely related to the average local kinetic temperature [36]. Sufficient conditions for the constancy everywhere of 〈ϕ(x,t)〉 are that, on the one hand, $\langle \left(x_{n+1}-x_{n}\right)\phi_{n}\rangle =\langle x_{n+1}-x_{n}\rangle \langle \phi_{n}\rangle$, and on the other hand that $\langle {\dot{x}}_{n}\;\;e_{n}\rangle =0$. The former condition implies that the conductive contribution to the energy flux is a constant multiplied by the average local inter-particle distance, which supplemented by the latter condition on the convective contribution implies the constancy everywhere of 〈ϕ(x,t)〉. This “separable” regime has been suggested to hold at low temperatures [33]; however, it may additionally depend on the temperature gradient, at least in principle. If, however, $\langle \left(x_{n+1}-x_{n}\right)\phi_{n}\rangle \neq \langle x_{n+1}-x_{n}\rangle \langle \phi_{n}\rangle$, then the constancy in space of 〈ϕ(x,t)〉 may be related to some nonuniformity of the convective term, $\langle {\dot{x}}_{n}\;\;e_{n}\rangle$. As an example, suppose that $\langle \left(x_{n+1}-x_{n}\right)\phi_{n}\rangle$ were a constant in *n*, in spite of $\langle x_{n+1}-x_{n}\rangle$ being not. Then, either 〈ϕ(x,t)〉 is not uniform in space, or $\langle {\dot{x}}_{n}\;\;e_{n}\rangle$ varies with *n*. In the following two sections, we shall try to elucidate one perspective which could potentially resolve this apparent paradox.

## 7. One Possible Attempt at a Resolution

One formal attempt at resolving some of the mentioned issues is to adopt a model of h(x,t), which gets rids of the most inconvenient singularities while still preserving a consistent description of the distribution of energy. One (not unique) such possibility is to define

(75) $$h\left(x,t\right)=\sum_{n}{}^{\prime }\frac{\epsilon_{n}R_{n}}{X_{n}}\;.$$

Thus, if, for example, $x_{n}<x<x_{n+1}$, then $h\left(x,t\right)=\epsilon_{n}/X_{n}$, i.e., the energy (potential plus average of the two kinetic energies) is uniformly distributed in the interval $\left(x_{n},x_{n+1}\right)$, and in such a way that $I_{x_{n},x_{n+1}}\;\;h=\epsilon_{n}$. Locally, the energy density is non-zero almost everywhere. There are discontinuities in h(x,t), i.e., it is a piecewise function, with no delta-type singularities. When a particle crosses the position at *x*, the value of h(x,t) jumps (by a finite amount). Our aim is to obtain a form of ϕ(x,t) which keeps track of the crossing of points held fixed in time, and thus of transport of matter, in some way which is more easily handleable in simulations.

After some algebra, we find

(76) $$\phi \left(x,t\right)=\sum_{n}\left(\frac{{\dot{\epsilon }}_{n}}{X_{n}}\left(x_{n+1}-x\right)+\phi_{n}^{†}+\frac{\epsilon_{n}}{X_{n}^{2}}\left[{\dot{x}}_{n}\left(x_{n+1}-x\right)+{\dot{x}}_{n+1}\left(x-x_{n}\right)\right]\right)\;\;R_{n}\;,$$

with ${\dot{\epsilon }}_{n}$ and $\phi_{n}^{†}$ given by Equations (60) and (44), respectively.

Via Equation (76), it is thus possible to assign a computable value (and its time average) for every position in the line. It is not guaranteed that such a flux would be uniformly distributed along (0,L), which is possibly related to the nonuniform density which is often seen in simulations [36]. Nevertheless, the contribution to the energy flux due to transport is now naturally included, albeit in a nonstandard definition of the energy density. While $\left(d/dt\right)\;\;I_{x_{n},x_{n+1}}\;\;h=\phi_{n-1}^{†}-\phi_{n}^{†}$ holds, we also have,

(77) $$\int_{x_{n}}^{x_{n+1}}\frac{\partial h(x,t)}{\partial t}\;dx=-\int_{x_{n}}^{x_{n+1}}\frac{\partial \phi (x,t)}{\partial x}\;dx=-\left(\phi_{n}^{*}-\phi_{n-1}^{*}\right)\;,$$

(78) $$\phi_{n}^{*}=\phi_{n}^{†}+\frac{1}{2}\;\;{\dot{x}}_{n+1}\left(\frac{\epsilon_{n}}{X_{n}}+\frac{\epsilon_{n+1}}{X_{n+1}}\right)\;.$$

Therefore, Equation (46) is not satisfied, and the effective primitive of ϕ(x,t) under the operator $I_{x_{n},x_{n+1}}$ is the usual interaction contribution $\phi_{n}^{†}$, plus a transport-type term. In loose terms, via our new definition (75), transport of matter is indirectly monitored by the modulation contributed by the denominator, $X_{n}=x_{n+1}-x_{n}$. Also recall that for delta-type energy density, often the effective primitive could not be defined, because of singularities at the extremes of integrations, which are now removed.

Equation (78) is another candidate for new investigations with numerical simulations of the relevant models. Finally, notice that

(79) $$\begin{array}{cc} I_{x_{n},x_{n+1}}\phi =\int_{x_{n}}^{x_{n+1}}\phi \left(x,t\right)\;\;dx=\frac{1}{2}\left(x_{n+1}-x_{n}\right)\;\;\left(\phi_{n-1}^{†}+\phi_{n}^{†}\right)+\frac{1}{2}\;\;\left({\dot{x}}_{n}+{\dot{x}}_{n+1}\right)\;\;\epsilon_{n}\;,\; & \end{array}$$

which is closely similar to Equation (62).

This leads us to the main proposition of the next section. Divide Equation (79) by $X_{n}=x_{n+1}-x_{n}$:

(80) $$\frac{I_{x_{n},x_{n+1}}\phi }{X_{n}}=\frac{1}{2}\;\;\left(\phi_{n-1}^{†}+\phi_{n}^{†}\right)+\frac{1}{2}\;\;\left({\dot{x}}_{n}+{\dot{x}}_{n+1}\right)\;\;\frac{\epsilon_{n}}{X_{n}}\;.$$

Then, by summing over *n* both Equations (78) and (80), we get

(81) $$\sum_{n}{}^{\prime }\phi_{n}^{*}=\sum_{n}{}^{\prime }\;\;\frac{I_{x_{n},x_{n+1}}\phi }{X_{n}}\;,$$

or, more explicitly,

(82) $$\sum_{n}{}^{\prime }\int_{x_{n}}^{x_{n+1}}\frac{\partial \phi (x,t)}{\partial x}\;dx=\sum_{n}{}^{\prime }\frac{\int_{x_{n}}^{x_{n+1}}\phi \left(x,t\right)\;\;dx}{x_{n+1}-x_{n}}\;.$$

## 8. Proposition for a Novel Definition

Equation (82) relies on a peculiar model for the energy density, i.e., Equation (75). It is a strong result, in that it does not rely on taking the time averages of the relevant quantities, but it holds instantaneously, equating the total in and out flow at the boundaries to the sum of the locally integrated fluxes, each divided the inter-particle distances. Equation (82) suggests the following interpretation, which we conjecture it may eventually apply to the summands terms, rather than merely to their sum. $I_{x_{n},x_{n+1}}\phi$ is the integrated flux between $x_{n}$ and $x_{n+1}$. As we have seen repeatedly, by allowing the extremes of integration to be time-dependent, this integral quantity shows to capture both the flux due to the interaction (conduction) and that which is usually associated to transport (convection). At the same time, it measures the integral flux between the two particles, and therefore over a (time-varying) distance $X_{n}=x_{n+1}-x_{n}$. Such separation is a microscopic distance, i.e., it is the smallest scale over which variations of the considered physical observables take place. Akin to taking a coarse-grained first derivative, we therefore assume that the total flux associated with the segment $x_{n+1}-x_{n}$ is given by $I_{x_{n},x_{n+1}}\phi$ divided by $x_{n+1}-x_{n}$ itself, somewhat echoing the mean value theorem. In general, we suggest the following form for the total flux pertaining to the *n*-th and (n+1)-th particles, which we label $\Phi_{n}$, independently of the specific definition which one assumes for h(x,t),

(83) $$\Phi_{n}=\frac{I_{x_{n},x_{n+1}}\phi }{X_{n}}=\frac{\int_{x_{n}}^{x_{n+1}}\phi \left(x,t\right)\;\;dx}{x_{n+1}-x_{n}}$$

which in turn typically translates into expressions of the type

(84) $$[\mathrm{Total}\;\mathrm{flux}\;\Phi {]}_{n}=[\mathrm{Interaction}\;\mathrm{flux}\;\phi {]}_{n}+\frac{{\left[\text{Integral}\;\text{flux}\;\text{transport}\right]}_{n,n+1}}{x_{n+1}-x_{n}}$$

and, more specifically, denoting CoM for Center of Mass, indexing with *n*, the segment n,n+1 is

(85) $$\Phi_{n}={\left[\text{CoM}\;\text{Velocity}\right]}_{n}\;\times \;{\left[\mathrm{Force}\right]}_{n}+\frac{{\left[\text{CoM}\;\text{Velocity}\right]}_{n}\;\times \;{\left[\mathrm{Energy}\right]}_{n}}{x_{n+1}-x_{n}}$$

The first term on the right hand side of Equation (85) may be replaced by an expression of the type ${\left[\mathrm{Velocity}\right]}_{n}\;\times \;{\left[\mathrm{Mean}\;\mathrm{Force}\right]}_{n}$, while the latter term on the right hand side of Equation (85) may be replaced by an expression of the type $\frac{[\mathrm{Mean}\;\mathrm{of}(\mathrm{Velocity}\;\times \;{\mathrm{Energy)})]}_{n}}{x_{n+1}-x_{n}}$, depending on the specific model of h(x,t) which is being utilized. In fact, as we have appreciated, there is some degree of arbitrariness in establishing the amount of energy density that one should allocate to each point in space. Just as two closely related examples, alternative to the formula of Equation (80), for the total energy flux $\Phi_{n}$ we propose

(86) $$\Phi_{n}=\phi_{n}^{†}+\frac{1}{2}\;\;\frac{\left({\dot{x}}_{n}+{\dot{x}}_{n+1}\right)\;\;\epsilon_{n}}{x_{n+1}-x_{n}}$$

(87) $$\Phi_{n}=\phi_{n}+\frac{1}{2}\;\;\frac{{\dot{x}}_{n}\;\;e_{n}+{\dot{x}}_{n+1}\;\;e_{n+1}}{x_{n+1}-x_{n}}$$

We were unable to establish, in rigorous terms, whether the above definitions are all consistent with each other for NESS’s, nor whether in general $\langle \Phi_{n}\rangle =\langle \phi_{n}\rangle$. Indeed, although Equation (77) shows that the considered integral can be expressed as a difference operator, we nevertheless could not devise a proof that that integral is zero in stationary states, therefore that $\langle \phi_{n}^{*}\rangle =\langle \phi_{n-1}^{*}\rangle$. We can therefore merely conjecture a stronger statement, and a weaker statement. The stronger statement asserts the independence on *n* of

(88) $$\langle \Phi_{n}\rangle =\langle \frac{\int_{x_{n}}^{x_{n+1}}\phi \left(x,t\right)\;\;dx}{x_{n+1}-x_{n}}\rangle$$

The weaker statement is the independence on *n* of

(89) $$\frac{\langle j_{n}\rangle }{\langle x_{n+1}-x_{n}\rangle }=\frac{\langle \int_{x_{n}}^{x_{n+1}}\phi \left(x,t\right)\;\;dx\rangle }{\langle x_{n+1}-x_{n}\rangle }$$

In either case, the averaged quantities should be confronted with $\langle \phi_{n}\rangle =\langle \phi_{n}^{†}\rangle$. At least for the latter case, already suggested by A. Politi (see acknowledgemnts in [35]), there is a reasonable expectation of its validity on physical grounds. Say 〈φ〉 is the NESS average of the “real”, physical energy flux φ at any given arbitrary point on the line (of which the chosen ϕ=ϕ(x,t) is a model). Even if the density is not uniform, one expects $\langle I_{x_{n},x_{n+1}}\phi \rangle =\langle x_{n+1}-x_{n}\rangle \langle \varphi \rangle$ to hold, i.e., accumulation of energy can only be transitory. Conversely, as for the statement in Equation (88), at least limited to the choice of the model of Equation (76) for ϕ(x,t), which is sufficiently regular, one notices that a purported proof may consider to equate both terms of Equation (88) to $\langle \phi \left(\xi_{n}\left(t\right),t\right)\rangle$, with $\xi_{n}\in \left[x_{n},x_{n+1}\right]$; then, to prove that $\langle \phi \left(\xi_{n}\left(t\right),t\right)\rangle =\langle \varphi \rangle$. The fact that the equality holds for a stationary $\xi_{n}$ is insufficient as a proof for the law $\xi_{n}\left(t\right)$.

Via numerical simulations, one could eventually establish whether all of the above expressions are consistent with the requirement that the energy flux be uniformly distributed in space, rather than only uniformly distributed among the particles. Recall that we also proposed Equation (76) as a test formula to verify whether or not, for the chosen model of energy density of Equation (75), the energy flux turns out to be uniformly distributed in space, in spite of the density profile being not.

## 9. Conclusions

We have analyzed the impact of changing the definition (model) of the energy density of a linear chain of particles with conserved positional order on the definition of energy flux. Several models of energy density can be proposed, all consistent with the requirement that they output the correct energy attributable to the particles, once the density is integrated over the particles’ separations. Among the several abstract possibilities, we have considered explicitly the (traditional) case of an energy density which is delta-distributed, one which is piecewise distributed, and one hybrid of both. Consistent with the existing literature, we considered two types of contributions to the energy flux: one which we called conductive and one called convective. We showed that the two contributions can be singled out by integrating the energy flux over properly defined time-dependent extremes of integration, tracking the particles’ positions in time. We found that the conductive contribution, in its discrete variant, is strong against variations of the definitions of the energy density, at least when NESS averages are considered. The integrated flux adds the convective term to the discrete variant of the energy flux, however we could not find an argument ensuring that this quantity is constant on average along the chain of particles. Furthermore, contrarily to the discrete version of the conductive term, the convective term seems to be more sensitive to the definition that one assumes for the energy density.

We have also shown that, in the framework of Fourier space for a delta-distributed energy density, the discrete version of the conductive contribution is exact in the k=0 wave-number limit, suggesting that it dominates the energy flux for infinitely extended chains. Our formula is also exact for all values of *k*, differently from previous propositions which relied on low wave-number expansions.

As the integrated flux is not straightforwardly connected to the local heat flux, we have proposed that a better candidate for identifying the total (conductive and convective) energy flux is the locally integrated flux, normalized by the instantaneous inter-particle separation. More specifically, we have shown that for one specific model of the (now piecewise) energy density, this identification can be constructed analytically. The piecewise energy density is in principle computable from simulations for any coordinate *x*, therefore it grants the possibility to attribute a flux value to any coordinate in space. In turn, such value can be directly confronted with the contribution to the flux that one obtains by considering the energy variations of the moving particles (which are not stationary, if not on average). A coherent picture shall emerge if the chosen model and formalism are indeed both correct and consistent, and this field of study is now open to future investigations.

It remains an open question how the above considerations can be adapted to linear models whose particles’ ordering on the line is not conserved over time, e.g., in the absence of a diverging contact repulsion. Furthermore, it would be interesting to investigate whether our proposed construction is sufficient to elucidate the extent to which the energy flux corresponds to heat flux in real physical systems. We hope that the present formalism may also be extended to three dimensions, a problem which has not been pursued here. In fact, the presented Fourier analysis can be now reinterpreted in the proposed new framework, and Fourier analysis is amenable to generalizations in more dimensions.
